# Modeling the volume of tissue activated in deep brain stimulation and its clinical influence: a review

**DOI:** 10.3389/fnhum.2024.1333183

**Published:** 2024-04-10

**Authors:** Erin E. Patrick, Chance R. Fleeting, Drashti R. Patel, Jed T. Casauay, Aashay Patel, Hunter Shepherd, Joshua K. Wong

**Affiliations:** ^1^Department of Electrical and Computer Engineering, University of Florida, Gainesville, FL, United States; ^2^College of Medicine, University of Florida, Gainesville, FL, United States; ^3^Department of Neurology, Fixel Institute for Neurological Diseases, University of Florida, Gainesville, FL, United States

**Keywords:** volume of tissue activated, VTA, deep brain stimulation, DBS, neuroimaging, probabilistic stimulation atlas, connectivity maps

## Abstract

Deep brain stimulation (DBS) is a neuromodulatory therapy that has been FDA approved for the treatment of various disorders, including but not limited to, movement disorders (e.g., Parkinson’s disease and essential tremor), epilepsy, and obsessive-compulsive disorder. Computational methods for estimating the volume of tissue activated (VTA), coupled with brain imaging techniques, form the basis of models that are being generated from retrospective clinical studies for predicting DBS patient outcomes. For instance, VTA models are used to generate target-and network-based probabilistic stimulation maps that play a crucial role in predicting DBS treatment outcomes. This review defines the methods for calculation of tissue activation (or modulation) including ones that use heuristic and clinically derived estimates and more computationally involved ones that rely on finite-element methods and biophysical axon models. We define model parameters and provide a comparison of commercial, open-source, and academic simulation platforms available for integrated neuroimaging and neural activation prediction. In addition, we review clinical studies that use these modeling methods as a function of disease. By describing the tissue-activation modeling methods and highlighting their application in clinical studies, we provide the neural engineering and clinical neuromodulation communities with perspectives that may influence the adoption of modeling methods for future DBS studies.

## 1 Introduction

Deep brain stimulation (DBS) is a neuromodulatory therapy that has been used to treat various neurological disorders for over 40 years. DBS was first introduced throughout the late 1980s and 1990s by Benabid and colleagues as an alternative to lesional surgery for treating medication refractory Parkinson’s disease (PD) (Benabid et al., 1989, 1991, 1996, 1998; Benazzouz et al., 2000). In 1997, the FDA approved the use of thalamic DBS to treat PD tremor and essential tremor (Aum and Tierney, 2018) and the adoption of neuromodulation for movement disorders spread quickly soon after. Following its success in treating motor symptoms, DBS was studied for its utility in treating psychiatric disorders, beginning in 1999 when Nuttin and colleagues examined the impact of DBS in four patients with obsessive-compulsive disorder (OCD) (Nuttin et al., 1999). Over the last few decades, the FDA further approved the use of DBS for OCD and epilepsy (Nuttin et al., 1999). Currently, researchers are exploring the use of DBS for other psychiatric disorders, such as Tourette syndrome, major depressive disorder, eating disorders, substance use and addiction, chronic pain, tinnitus, Alzheimer’s disease, and anxiety disorder (Lee et al., 2019).

DBS is rapidly growing as a neuromodulatory therapy for many medication-refractory neurological diseases and it is estimated that by 2019 over 160,000 people had received DBS world-wide with a projected growth of more than 12,000 new implants per year (Lee et al., 2019). DBS can be very effective in select patients with fewer adverse events compared to traditional lesional procedures. DBS has progressively entered the clinical sphere as a predominant and effective solution for medication-resistant and refractory motor and psychiatric conditions (Mayberg et al., 2005; Greenberg et al., 2006; Schlaepfer et al., 2008; Malone et al., 2009; Parastarfeizabadi and Kouzani, 2017).

Methods to optimize the clinical benefit of DBS for individuals with various diseases are a major topic of current research in the field of brain neuromodulation therapy. Data-driven models made from retrospective studies of populations of patients are beginning to allow for more precise clinical guidance on surgical placement of DBS leads and programming of stimulation parameters of the electrodes (Wong et al., 2020). However, as more practitioners adopt the available methods, it becomes increasingly important for the community to understand their limitations and assumptions. The modeling tools including those that predict the extent of tissue activated by the stimulation (i.e., volume of tissue activated (VTA)) and its corresponding use in neuroimaging procedures that make it relevant to disease target structures or connected networks fall within this scope. By contextualizing VTA in the history of neuromodeling with respect to DBS and by analyzing how it works, we can demystify its function in an attempt to improve understanding for use in future clinical studies and practices. Similarly, by analyzing instances in which VTA with neuroimaging has been used clinically, we may extract its use in the progression of DBS therapy and identify areas in which advances in modeling are needed.

### 1.1 Overview of the VTA and its use with neuroimaging

The VTA approximates the spatial extent of the modulatory influence of a voltage field arising from stimulating electrode/s on neighboring neural tissue. It is visualized as a volume and is a useful metric with which to compare overlap with specific anatomical brain structures or fiber tracks connecting distant brain regions. This volume does not accurately represent the microscopic biophysical reality of cell-specific activation near the DBS lead; however, it has become a very useful tool for the study and prediction of clinical impact of DBS therapy. How the volume is calculated and the differences that lie there-in are discussed in Section 2.

The utility of the VTA is dependent on imaging the brain. Wårdell et al. provide a detailed review of imaging and modeling technologies for DBS (Wårdell et al., 2022). In short, the DBS lead location with respect to anatomical structures of the individual must be identified with pre-operative MRI for anatomy and post-operative MRI or CT scans for lead localization. Existing neurological atlases then need to be warped to the patient’s brain images to provide spatial reference points (Wårdell et al., 2022). Additionally, pre-operative MRI scans with diffusion tensor imaging (DTI) provide extra information that can be used to define local anisotropic tissue electrical conductivity values that can inform the VTA calculation (Tuch et al., 2001; Butson et al., 2007). This additional specificity can make for a more accurate VTA estimation, especially when patient-specific data is used (Malaga et al., 2021, 2023). However, DTI methodological parameters including voxel size and resolution can introduce error (Rodrigues et al., 2018). For example, when using VTA models that do not assume a uniform and homogenous tissue, the acquisition quality and parameters of the DTI sequences can impact the resulting distribution of the electric field and ultimately the shape of the VTA.

#### 1.1.1 Target-based stimulation mapping

**Figure 1:**
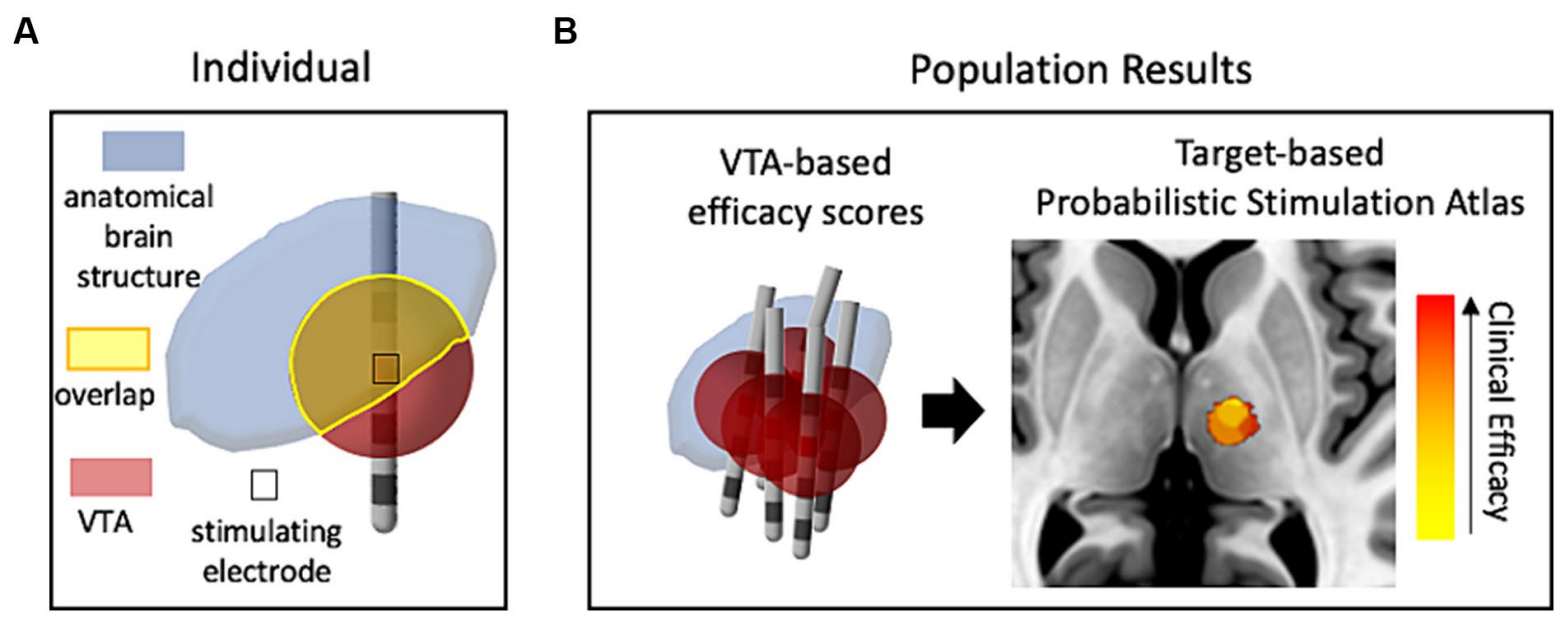
The use of VTA and structural neuroimaging in clinical retrospective studies. **(A)** Conceptual drawing of one patient’s VTA calculation compared (voxel-wise) with a neuroanatomical atlas to estimate the influence of stimulation on target structures. **(B)** Representative probabilistic results from a population of patients that show correlation of VTAs with clinical outcomes (efficacy of treatment with minimal side-effects). Red denotes high correlation with clinical efficacy while yellow denotes low correlation. The image of the brain was taken from standard template image (MNI 152 brand).

The initial use of VTA calculations, besides its role in the design and characterization of electrode/lead geometries (Butson and McIntyre, 2005b), was to determine to what extent target brain structures were affected by the electrical stimulation through viewing the overlap between the predicted VTA and the anatomical structure (McIntyre et al., 2004b), as depicted in Figure 1A. This vein of research has evolved into using data-driven approaches (Figure 1B), where populations of patient data provide probabilistic estimates of DBS efficacy and are used to help define optimal “sweet spots” for stimulation targets (Butson et al., 2011; Cheung et al., 2014; Eisenstein et al., 2014; Dembek et al., 2017; Reich et al., 2019). Different approaches have been used to cluster the patient data for development of probabilistic maps of stimulation efficacy, including using thresholding or voxel-wise statistical methods. Dembek et al., suggest that voxel-wise statistics that base outcome in a certain voxel against average clinical outcomes is a promising method in that it provides the most consistent results for different scenarios (Dembek et al., 2022). Other work highlighting the influence of nuances in methods within voxel-wise statistical approaches (Nordin et al., 2022) shows that this facet of DBS research is also still being refined. Probabilistic stimulation maps for prospective use in which an individual’s predicted VTA may be tuned via stimulation parameter selection for optimal mapping to a disease-specific probabilistic stimulation atlas will enable optimization for stimulation programming and lead placement and may reduce the amount of clinical trial and error.

#### 1.1.2 Network-based stimulation mapping

**Figure 2:**
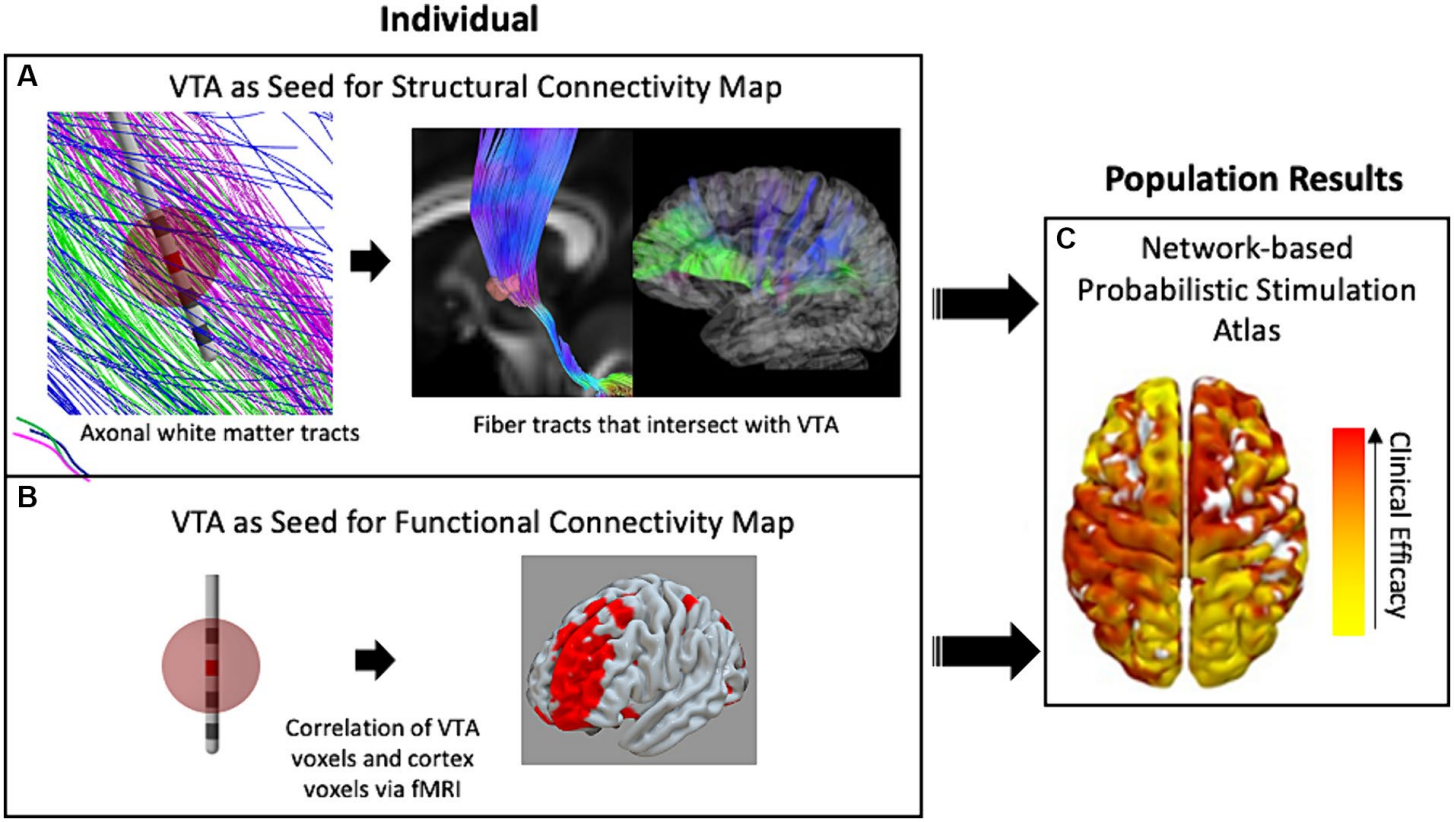
The use of VTA and connectivity mapping in retrospective studies. **(A)** White-matter fiber tracts intersecting the VTA enable estimation of connectivity with distant brain regions including the cortex. **(B)** The fMRI voxels included in the VTA are correlated with whole-brain fMRI to map functional connectivity in a single individual. **(C)** Either technique for connectivity mapping may be used in retrospective studies of populations of patient groups to result in a data-driven network-based connectivity atlas for a specific disease. Red denotes high correlation with clinical efficacy while yellow denotes low correlation.

Alternatively, to get a better understanding of the underlying mechanisms of DBS with respect to network activity, researchers introduced techniques of using the VTA to seed calculations that determined which distant brain regions were either structurally or functionally connected to the stimulation site. For example, DTI information can be used to reconstruct and approximate axonal fiber tracts that reveal *structural* network connectivity throughout the brain (Horn et al., 2014). In this method, representations of individual fiber tracts that pass through the VTA are identified and traced to cortical or other distant brain areas (Figure 2A). Additionally, another method correlates the blood-oxygen-level-dependent (BOLD) brain signal at the voxel level via whole brain functional MRI (fMRI) with voxels within the VTA to identify distant regions that are *functionally* connected to the VTA area (Al-Fatly et al., 2019). The two methods are conceptually depicted in Figure 2. Both methods may be used within a population study to result in independent network-based stimulation atlases/maps. VTAs can also be used to compute these network metrics at a cohort level within a framework known as a “connectome.” A connectome represents group-averaged structural or functional connectivity data that can either be patient-and disease-specific or normative—meaning a standardized dataset generated from a large collection of healthy volunteers.

In summary, VTA computations in conjunction with advanced neuroimaging has allowed for much insightful information to be gleaned from population-based retrospective studies. The field is on the verge of being able to use the results of these studies to perform clinical predictions that could aid in the presurgical planning of lead placement and reduce programming time after implantation. This technology will enable clinician guidance that is individualized to each patient. However, as precision is necessary for patient-specific applications, the errors and assumptions within the modeling methods need to be fully understood. In this review, we highlight key differences in the methodologies and biophysical foundations of current VTA models (Section 2), identify the VTA/neuroimaging models used in a collection of disease-specific clinical studies (Section 3), and comment on trends and use-cases (Section 4). The errors and biases found in the methods for target-or network-based stimulation mapping are not discussed in detail in this review.

## 2 Calculating the VTA

**Figure 3:**
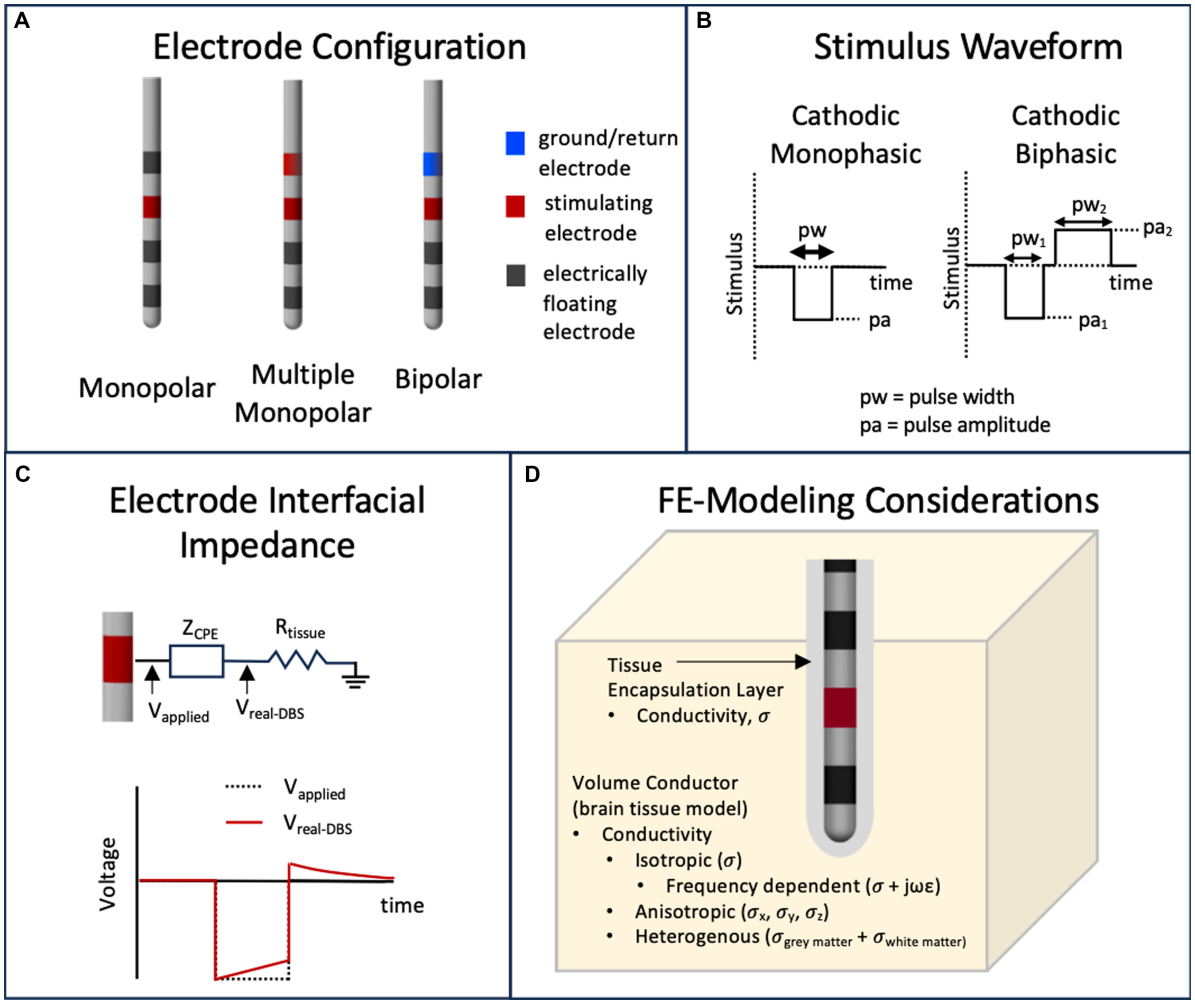
Parameters that influence the calculation of the VTA. **(A)** Typical DBS leads can have various stimulating electrode configurations including monopolar, bipolar and multimonopolar. **(B)** Typical stimulus waveforms consist of cathodic monophasic or cathodic biphasic voltage controlled or current controlled pulses with user defined pulse amplitude and pulse width. **(C)** The capacitive nature of the electrode interface, usually modeled as capacitor or a constant phase element (CPE) will filter a voltage-controlled stimulus pulse. **(D)** The volume conductor in a finite element model can have materials that model the brain tissue and tissue encapsulation layer with various representations of electrical conductivity.

DBS stimulation hardware provides rectangularly shaped stimulus waveforms, which may be voltage or current-controlled (Amon and Alesch, 2017). However, traditionally, voltage-controlled waveforms have been used clinically and thus many of the VTA approximation methods are based on voltage input parameters. The waveform is in the form of pulse trains with pulse width on the scale of tens to hundreds of microseconds and pulse frequency in the low hundreds of Hz (Volkmann et al., 2006). These stimulation (or programming) parameters including pulse shape (such as monophasic or biphasic) are needed to define the VTA around one or multiple stimulating electrodes. Frequency has been shown to have minimal effects on the physics-based calculation of the VTA (Duffley et al., 2019) and thus is usually neglected in VTA estimation methods. Different electrode configurations such as monopolar (i.e., one stimulating electrode with respect to a distant ground electrode), bipolar (one stimulating electrode with respect to an adjacent ground electrode), or multiple monopolar are also possible. Figures 3A,B capture these parameters. Figure 3A shows examples of monophasic and biphasic stimulus waveforms with negative (cathodic) leading pulses and Figure 3B shows examples of monopolar or bipolar electrode configurations on a typical DBS lead (such as the Medtronic 3387 lead) with four cylindrical electrode contacts. In the monopolar configurations, the ground contact is the internal pulse generator (IPG) and is usually modelled as the outer boundary in FEM simulations (Butson and McIntyre, 2005b; Aström et al., 2009).

Existing research-grade, commercial, and open-source simulation toolboxes that couple the VTA estimations with neuroimaging data employ a variety of methods to compute the VTA. As a result, the term “VTA” has taken on several definitions across the literature and commercial platforms. Each VTA model has inherent assumptions and limitations, which may make one model more appropriate than another for certain clinical DBS studies. In this section, we will highlight the major assumptions and limitations for the major VTA approaches; but first we present historical context with respect to modelling neural activation and DBS.

### 2.1 Modeling neural activation—historical context

Computational models used to predict neural excitation have evolved considerably since 1907 when Louis Lapicque first described the integrate-and-fire model (IF) as a tool to simulate the spiking activity of neuronal membranes using a parallel capacitor and leak resistor (Abbott, 1999). Hodgkin and Huxley provided a more complex equivalent circuit model of the cell membrane of the squid giant axon that incorporated the dynamics of specific ion-channels constituting the action potential (Hodgkin and Huxley, 1952). Incorporating a Hodgkin and Huxley-style membrane model, Wilfrid Rall developed a mathematical model by which to simulate the electrophysiology of realistic morphologies of neurons including the soma, dendritic tree, and axon (Rall, 1962). Using cable and core-conductor theory (Rall, 2011), his work set the stage for multi-compartment cable models of neurons and axons that are widely used today in computational neuroscience.

Regarding DBS, multi-compartment cable models were first used to try to elucidate the therapeutic mechanism of action of the stimulation. Four theories were tested with biophysically accurate models of thalamocortical relay neurons (including soma, dendrites, and axon) (McIntyre et al., 2004a). Of the four theories, three hypothesized that electrical stimulation inhibited neuronal responses through (1) blockade of voltage-gated currents in the neurons, (2) synaptic inhibition of neurons, or (3) synaptic transmission failure due to transmitter depletion. The fourth theory suggested that electrical stimulation directly modulated the pathological network activity via modulation of surrounding neural tissue—an informational lesion. McIntyre et al. showed that physics-based computational modeling results compared with functional imaging and neural recording suggest that the fourth theory is most probable (McIntyre et al., 2004a,c). However, there is still much ambiguity in the biological mechanisms that underlie the effectiveness of DBS for various diseases (Lee et al., 2019). Nevertheless, computational modeling studies also showed that the axon (or “fiber of passage”) was most important to model since it could be most easily depolarized by electrical stimulation and was the primary conduit for action potential propagation (McIntyre et al., 2004a,c). Based on these findings, researchers began to use modeling to predict an approximated volume of tissue activated (VTA) surrounding the active DBS contact as a function of stimulation parameters. Since biophysically accurate, physics-based models are computationally expensive and require specific computing skills, methods to calculate a VTA manifested in a myriad of flavors with different levels of approximations.

### 2.2 FEM-based models

**Figure 4:**
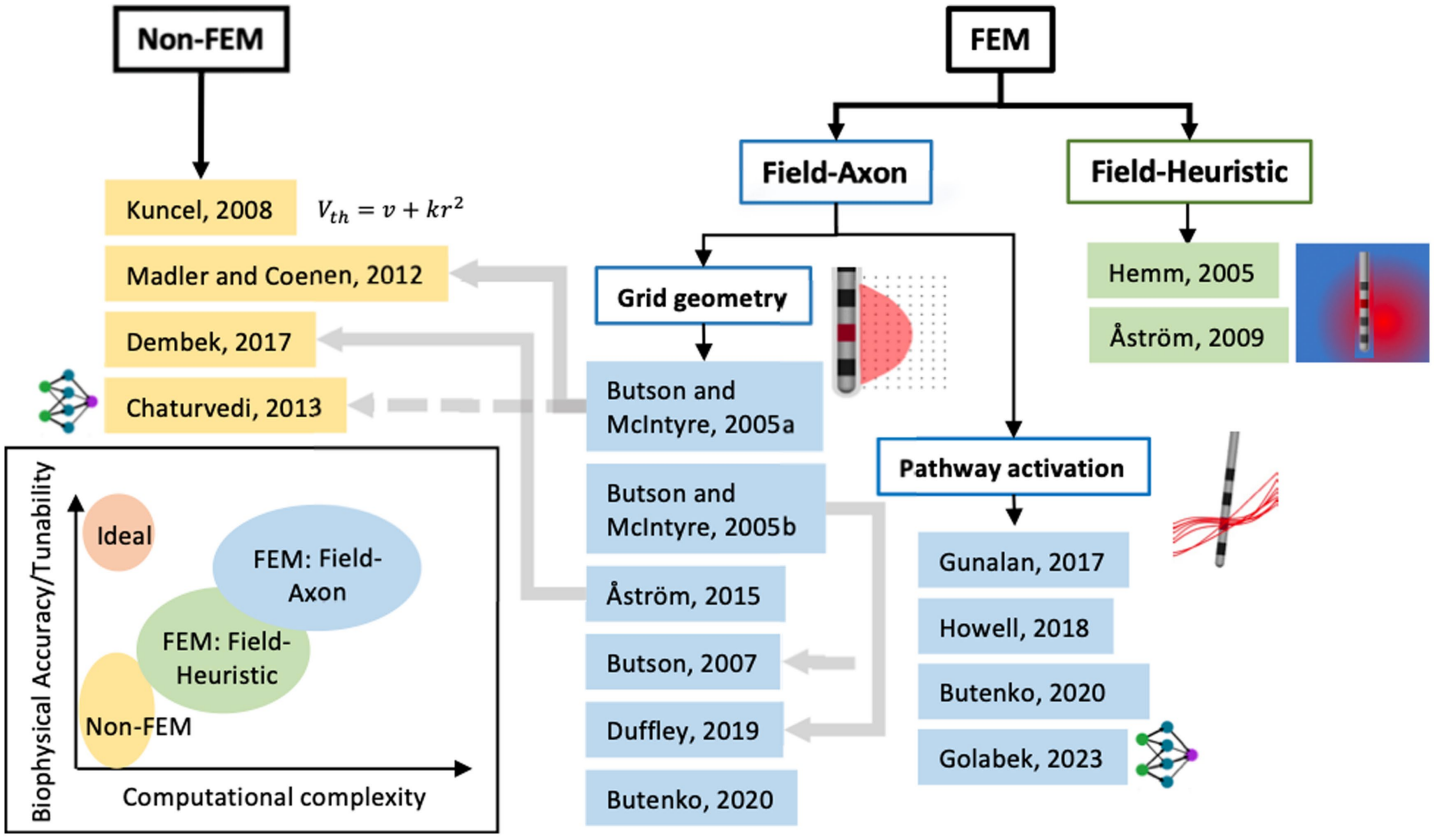
Classification of seminal papers on modeling methods for volume of tissue activated or pathway activation based on using finite-element methods (FEM) or not. Further classification can be made with axon biophysical models (field-axon) or not (field-heuristic) and then an even further classification between axons placed on a grid in conventional VTA and axons defined by tractography. The grey lines denote dependence of one model on the former. The groups of models can be assigned levels of computational complexity and biophysical accuracy or tunability as seen in the chart (bottom left).

Generally, a top-level distinction can be drawn between VTA methods that either need an extra computational tool that uses the finite element method (FEM) to compute the voltage field and/or its spatial derivatives as input to its VTA model, or ones that do not (Figure 4). The papers referenced in Figure 4 and described in Sections 2.1–2.3 are the seminal papers describing new VTA methods or new increments to known methodologies; it is not intended to be comprehensive of all papers published on VTA methods.

The finite-element method is commonly used to numerically solve for the voltage field, V, resulting from a stimulating electrode in a 3-dimensional conductive medium defined by conductivitys σ, which can be real or complex to incorporate frequency dependence in a tissue model (i.e., capacitive effects). The differential equation solved by FEM tools to calculate the VTA is the Laplace equation:


(1) ∇⋅σ∇V=0,

with boundary conditions that reflect the stimulation voltage or current at electrode contacts. FEM-based models are dependent on the physical parameters that incorporate the geometry of the DBS lead, tissue heterogeneity and anisotropy, and the presence of encapsulation tissue around the lead due to a foreign body immune reaction. Figure 3D depicts these parameters. Typically, these FEM simulations are time-independent and thus the time dependence of the voltage waveform (Figure 3A) or nonideal capacitive effects of the electrode/electrolyte interface (Brug et al., 1984; Butson and McIntyre, 2005a; Miocinovic et al., 2009) as seen in Figure 3C are not modeled in the FEM simulation. Time-dependent effects—the capacitively filtered stimulus waveform and the programming parameter pulse width—are usually taken into account using separate methods (e.g., incorporated into the equivalent circuit-based field-axon simulation), but can be incorporated in FEM simulations using Fourier methods; e.g., the OSS-DBS simulation framework has this capability (Butenko et al., 2020).

As a secondary distinction between VTA methods within the FEM branch (Figure 4), FEM-derived values of the voltage field are used to compute the VTA via heuristic models or ones derived from multicompartment axon models (i.e., field-axon). Field-heuristic models, discussed in the following section, couple a FEM solution of field values (i.e., 1st spatial derivative of the voltage field) with clinical data of DBS effectiveness.

#### 2.2.1 Field-heuristic

With least computational complexity out of the FEM-based models, field-heuristic VTA models use computational methods to define iso-contours or iso-surfaces of electric field values that correlate with perceived clinical efficacy. Hemm et al. first introduced this method to help visualize the extent of the VTA by correlating calculated 2D field contours with MR brain images and therapeutic DBS parameters in patients with leads in the internal globus pallidus (GPi) for dystonia therapy (Hemm et al., 2005a,b). They used a homogenous, isotropic tissue conductivity model and surmised that for numerous patients using monopolar, double monopolar, or bipolar electrode configurations, the absolute value of electric field vector of 0.2 V/mm was a good estimate of GPi coverage.

Aström et al. further evaluated this method of using the computed electric field in an FEM model and a heuristic threshold value of 0.2 V/mm to help visualize a VTA using a heterogenous tissue model (Aström et al., 2009). Their paper states that “[this method] should only be interpreted as a boundary of tissue, where the electric field (absolute value of the 3D vector) is 0.2 V/mm or larger, and not as the volume of tissue influenced by the stimulation,” but nonetheless was used to help explain stimulation-induced side effects of DBS for Parkinson’s disease therapy in one patient. The FEM model was used to compute the maximum electric field in 3D space with a heterogenous model space of white and grey matter ( σ = 0.06, 0.09 S/m, respectively) based off of patient-specific MRI data and with normalized stimulus voltages (reduced by a factor 0.89) to accommodate for non rectangular stimulus waveforms from the IPG (Butson and McIntyre, 2007), but no tissue encapsulation was modeled. The 0.2 V/mm threshold value was based off of two clinical inferences: (1) the isolevel of 0.2 V/mm was within a clinically effective radius of 2–5 mm in the STN as given by (Volkmann et al., 2006) for Parkinson’s disease therapy using conventional monopolar programming and (2) Hemm’s early work with calculated electric field values and GPi stimulation for dystonia (Hemm et al., 2005a,b).

Choosing a single electric field threshold to compute a volume is inherently dependent on the FEM model parameters (e.g., tissue conductivity, heterogeneity, etc.) and thus prone to large variability, plus that it does not take into account time dependency on stimulus waveforms. However, many clinical studies (referenced in Section 3) use the 0.2 V/mm electric field threshold as a VTA metric as it is a straight-forward method.

#### 2.2.2 Field-axon—grid geometry

The other types of FEM models couple field information to multicompartment models of myelinated axons. One subset of these models place axons in a grid perpendicular to the long axis of the DBS lead (i.e., grid geometry) and estimate a volume based on the extent of activation of individual axons at certain stimulation parameters (e.g., voltage, pulse width, and sometimes pulse train frequency and capacitive effects of the tissue or electrode interface that shape the waveform). Another subset of field-axon models, pathway activation models, make use of more realistic axon pathways via MRI-based tractography (Coenen et al., 2012) and will be discussed in more detail in Section 2.2.3.

Butson and McIntyre (2005a) developed the first of the grid-geometry field-axon models. Building on earlier work (McIntyre et al., 2004b), they performed a simulation study that used multicompartment models of a 5.7 mm diameter myelinated axon (McIntyre et al., 2002) to predict action-potential excitation from monopolar cathodic stimulation of a Medtronic 3387/3389 DBS electrode for a single pulse width of 90 μs over a range of isotropic tissue conductivities and encapsulation conductivities. Fibrotic tissue encapsulation was modelled in the FEM model as a space adjacent to the lead with a range of lower conductivities (by a factor of 2, on average) than the bulk tissue. In subsequent work (Butson and McIntyre, 2005b), Butson and McIntyre again used the same 5.7 mm myelinated axon grid-model to predict activation for multiple pulse-width cathodic monopolar signals in a homogeneous isotropic medium with conductivity equal to 0.3 S/m; however, their goal was to define an analytical equation fit to their simulation results that would decrease the time and computational complexity of VTA prediction. They make use of the activating function (Rattay, 1986), which is the second spatial derivative of voltage ( $\nabla^{2}V$ ), and shown to be a useful value to define a threshold since it is relatively constant over a range of electrode designs (Butson and McIntyre, 2005b). Their result was a response curve of activating function thresholds for the individually placed axons as a function of the product of stimulus amplitude and pulse width. Because of their axisymmetric simulation, a 2-D contour line based on an activating function value for specific stimulus voltage amplitude and pulse width parameters can be rotated around the lead to define the 3-D VTA. Their work instantiated a look-up-table (LUT) approach for fast prediction of VTAs based on stimulation parameters (pulse amplitude and width). The 2005b model was limited in that only isotropic media with conductivity of 0.3 S/m was used in the FEM, and thus does not apply in heterogeneous and anisotropic models. Cicerone (Miocinovic et al., 2007), an academic software coupling VTA with neuroimaging software that has been commercialized by Boston Scientific as GUIDE, uses precompiled data from simulations using methods from the above studies to provide fast computation for a number of monopolar electrode settings. The effects of electrode interfacial impedance (Butson and McIntyre, 2005a) are incorporated in Cicerone.

In an effort to compare electric field thresholds with activating function thresholds and to analyze the effect of other considerations such as fiber diameter, Åström et al. used multicompartment cable models coupled with FE modelling (Åström et al., 2015). Their model used slightly different biophysical parameters for the axon model than used by the Butson-McIntyre models and employed a single cable model instead of a double cable model; however, the fibers were similarly placed in a grid perpendicular to the DBS lead. Also, the FEM simulation used an isotropic conductivity of 0.1 S/m in the volume conductor. Their study suggested that isosurfaces generated by electric field thresholds and activating thresholds could be used to approximate the VTA defined by field-axon simulations. They also show that the electric field (1st spatial derivative of voltage) rather than the activating function (2nd spatial derivative) is a simplified metric by which to define VTA threshold values, supporting their earlier work (Aström et al., 2009) because it is largely independent of stimulation amplitude for large diameter fibers for a specific pulse width. Thus, their method when used for fast prediction, could also entail a look-up table approach with computation of the electric field to estimate the VTA for a stimulus of a specific pulse width, only. Of note, their results for a fiber diameter of 3.5 mm matched simulation results for the Butson-McIntyre 5.7 mm diameter model. The nodal spacing, which turned out to be a large factor in excitability, was similar for the individual axon models of the mentioned diameters. Two opensource packages use these results for fast computation. Lead DBS (Horn et al., 2014) and FastField (Baniasadi et al., 2020) compute VTAs from electric field isocontours based on data from Table 3 in Åström et al. (2015) for cathodic pulse widths of 30, 60, 90, and 120 μs and fiber diameters from 2 to 5 μm. In these cases the average electric field value, which corresponds to a voltage pulse amplitude of 3 V, is used as the VTA threshold metric.

One limitation of grid-geometry models is that isotropic tissue conductivity must be used since the volume is defined by revolving the contour created by individual fiber activation in the plane grid-geometry. Later work by Butson et al. using the activating-function threshold approach showed that these models can be adapted to accommodate anisotropic tissue conductivities, defined by DTI, and create 3D surfaces informed by the local conductivity (Butson et al., 2007). How to best compute the second spatial derivative for a 3D volume was the topic of the latter two studies (Anderson et al., 2018; Duffley et al., 2019). The max eigenvalue of the Hessian matrix, which is the spatial partial second derivative of voltage, is used in these models to define a VTA volume that is independent of fiber orientation. Being that it uses the max value along any spatial direction, it could overestimate activation for realistic fiber trajectories; however, it is a very convenient method to use with anisotropic or heterogenous conductivity in FEM models. The absolute value of the 3D electric field vector can similarly be used to define a VTA within a finite-element model with local conductivities defined by DTI data; its estimation could also lead to overestimation.

#### 2.2.3 Field-axon—pathway activation models

Pathway activation models (PAM) define the axon pathways, from either patient DTI-based tractography information or structural connectome and pathway atlas data sets, and are not technically VTA models, as activation of discrete fibers near the DBS electrode are computed rather than a volume. However, they do predict axon/fiber activation as a response of DBS and the methods used for these models fall under the field-axon classification (Figure 4). Gunalan et al. used detailed FEM models with multi-compartment axon models of 5.7 mm diameter to predict activation of axons in two corticofugal axonal pathways: the hyperdirect pathway and the internal capsule fibers of passage (Gunalan et al., 2017). Their modeling results correlated well with effective clinical stimulation settings in that a portion of the hyperdirect pathway fibers were activated and none of the fibers in the internal capsule were activated, which corresponded well with clinical hypotheses for efficacy.

Since multi-compartment axon models are computationally intensive, subsequent work by Howell et al. (2019) developed a linear approximation to the multi-compartment axon model that allowed for much faster prediction of FEM-informed pathway-activation models. They presented empirical models for a range of axon diameters. Additionally, Golabek et al. developed another pathway-activation fast-prediction method using an artificial neural-network model for the 5.7 mm diameter axon (Golabek et al., 2023).

Field-axon VTA or pathway activation models are ostensibly the most tunable for the specific application and thus can be made specific to patient MRI information, but the computational complexity can be a barrier to entry for many practitioners and clinicians.

### 2.3 Non-FEM-based models

Some VTA models do not need FEM computation as input. They are based on empirical clinical studies and/or rely on relationships derived from physics-based multicompartment axon models and thus are inherently specific to the data from which they were derived. However, because of not having to perform extra computation, these models are extremely fast; nevertheless, they may also introduce error to the study if used outside of their limits.

Kuncel et al. adapted an empirical model for neuron/axon activation that relates the threshold voltage to distance away from an electrode (threshold-distance relationship) for DBS for Parkinson’s disease (Kuncel et al., 2008). The empirical model, which was first determined for excitation of pyramidal-cell tracts in the cat motor cortex (Stoney et al., 1968) is described in Eq. 2. It assumes that the threshold voltage for excitation, $V_{th}\text{,}$ (which would represent the amplitude of the applied voltage pulse) is proportional (with constant k ) to the squared distance r from the electrode (which would represent the spherical radius of a volume of tissue activated) plus an offset v :


(2) $$V_{th}=v+kr^{2}\text{.}$$

Kuncel et al. tuned the empirical model based on paresthesia-related side effects of monopolar (cathodic) stimulation in the Vim nucleus of the thalamus. The Kuncel model is specific to monopolar stimulation on a model 3387 Medtronic DBS lead, a 90 μs cathodic monophasic voltage pulse and comprises an average of results from 8 subjects. Also, the influence of tissue and electrode impedance is inherently built into this model. Interestingly, as shown in Åström et al. (2015), the Kuncel model is equal to an electric field threshold value of 0.165 V/mm in isotropic media of 0.1 S/m.

Mädler and Coenen (2012) introduced another empirical model computing the VTA via a spherical radius from the middle of a monopolar DBS electrode as a function of stimulus voltage amplitude ( $V_{th})$ and electrode and electrode/tissue impedance by using the results of field-axon simulations in Butson and McIntyre (2005a). They fit a second order polynomial to the computational model results of the form:


(3) $$V_{th}\left(r,\Omega \right)=k_{0}+k_{1}r+k_{2}\Omega +k_{3}r^{2}+k_{4}\Omega +k_{4}\Omega^{2}\text{,}$$

where r is the radius from the center of the electrode, k ’s are fitting constants, and W is the impedance of the system. Impedance values from Butson and McIntyre (2005a) were determined from *in situ* impedance measurement by the IPG stimulator at a frequency of 30 Hz. Also, this model is specific to 90 μs cathodic monophasic pulse trains. According to Åström et al. (2015), this model is equivalent to an electric field threshold of 0.19 V/mm in isotropic media of 0.1 S/m when using an impedance of 1,000 Ohms, which is very close to the heuristic value of 0.2 V/mm.

Dembek et al. established another simple model to estimate the spherical radius, r , of VTA fit to Åström et al.’s (2015) field-axon simulations that incorporate the effect of pulse width on electric field threshold (Dembek et al., 2017). Their model which is derived from Coulumb’s law is as follows:


(4) $$r={\left(\frac{pw_{\mathrm{real}}}{pw_{\mathrm{given}}}\right)}^{0.3}*\sqrt{0.72\frac{I}{E_{\mathrm{given}}}}\text{,}$$

where I is an applied stimulus current amplitude, $pw_{\mathrm{real}}$ is the pulse width of the applied pulse, and $pw_{\mathrm{given}}$ and $E_{\mathrm{given}}$ are reference values of known correlation between pulse width and electric field threshold values. Dembek et al. used the values of 0.165 V/mm and pulse width of 90 μs for the “given” parameters that matched the empirical results from Kuncel et al. (2008) with the Åström et al. (2015) model for electric field threshold. If the impedance of the system is known (e.g., through measurement via the IPG), then a voltage amplitude instead of current may be used in this model. The Dembek et al. (2017) model generalizes monopolar, cathodic monophasic stimulation to user specified pulse widths to easily calculate a VTA.

The last of the non-FEM models is one by Chaturvedi et al. (2013), which uses large-scale computational simulation results stemming from the methods of Butson and McIntyre (2005a) and Miocinovic et al. (2007) to train an artificial neural network to predict VTAs based on numerous user input parameters. The parameters include stimulus voltage amplitude, pulse width, high, medium, or low tissue encapsulation resistance, and electrode configuration (4 monopolar, 18 bipolar configurations). The training data consisted of FEM results using isotropic tissue conductivity of 0.2 S/m outside of the tissue encapsulation layer. This is the only non-FEM model able to accommodate bipolar electrode configurations.

### 2.4 Open-source, academic, and commercial simulation platforms

Providing the community with simulation tools that couple neuroimaging with VTA or pathway-activation calculation with reduced computational burden has been the intent of commercial and open-source platforms. Table 1 lists these simulation tools and their base VTA model and associated available user input parameters. All of these platforms stem from the academic works discussed in Sections 2.2–2.3 and many use simplified algorithms based on precomputed data from detailed field-axon simulation studies. Moreover, this table lists what type of neuroimaging/visualization capability is paired with VTA simulation (i.e., anatomical target structures and structural and functional network connectivity) within each tool.

**Table 1:** Description of open-source, academic, and commercial VTA and neuroimaging visualization software platforms.

	Software platform	VTA method	Electrode type	Tissue/FEM parameters	Waveform parameters	Neuroimaging visualization with VTA
Open-Source	Lead DBS	Non-FEM: Kuncel et al. (2008), Mädler and Coenen (2012), Dembek et al. (2017)	Monopolar (Medtronic 3387, 3389)	See VTA method	Cathodic monophasic, 90 μs or variable pulse width; variable voltage amplitude	Anatomical target, structural network connectivity, functional network connectivity
		FEM: Field-heuristic or LUT based on Åström et al. (2015) data	Monopolar, bipolar	User-defined: variable heterogenous isotropic conductivity (grey/white matter)	Field-heuristic: voltage or current amplitude; LUT VTA parameters: cathodic pulse width 30, 60, 90, 120 μs, axon diameter (2–5 μm)	
		FEM: field-axon (Butenko et al., 2020)	User defined: any	User defined: any	User defined: any	
	FastField	FEM: Field-heuristic or algorithm based on Åström et al. (2015) data	Monopolar and multimonopolar for 12 electrodes from 4 different vendors	User defined: variable isotropic, homogeneous conductivity	Field-heuristic: voltage or current amplitude; LUT VTA parameters: cathodic monophasic, 30, 60, 90, or 120 μs pulse width, variable amplitude	Anatomical target
	ELMA+DBStim	FEM: Field-heuristic or LUT based on Åström et al. (2015) data	Monopolar, bipolar	Isotropic, heterogenous conductivity; with or without tissue encapsulation	Field-heuristic: voltage or current amplitude; LUT VTA parameters: cathodic pulse width 30, 60, 90, 120 μs, axon diameter (2–5 μm)	Anatomical target
	SciRun	FEM: field-axon (or Hessian matrix, Duffley et al., 2019)	User defined: any	User defined: any	User defined: any	May be combined with anatomical target and structural connectivity
Academic	Cicerone	Non-FEM LUT: based on Butson and McIntyre (2005b) and subsequent work	Monopolar (Medtronic 3387, 3389)	Electrode capacitance accounted for, isotropic homogenous tissue conductivity 𝜎=0.3 S/m, user adjustable: encapsulation impedance	Cathodic monophasic, user adjustable: voltage or current amplitude, pulse width, pulse frequency	Anatomical target
	StimVision v2	Non-FEM: based on Chaturvedi et al. (2013) FEM: pathway activation, Howell et al. (2019)	Monopolar, multipolar and multi-monopolar (MDT 3387, MDT 3389, ABT 6172, BSN 2202)	Homogeneous isotropic 𝜎=0.2 S/m, tissue encapsulation layer 𝜎=0.1 S/m	Cathodic monophasic with built in effect of electrode capacitance; user adjustable: stimulus amplitude, pulse width	Anatomical target, structural network connectivity
Commercial	SureTune 4 (Medtronic)	Non-FEM LUT: based on Åström et al. (2015) data	Monopolar	Homogeneous isotropic 𝜎=0.1 S/m	LUT VTA parameters: cathodic pulse width 30, 60, 90, 120 μs, axon diameter (2, 2.5, 3 μm)	Anatomical target, structural network connectivity
	Guide (Boston Sci.)	Non-FEM LUT: based on Butson and McIntyre (2005b) and subsequent work	Monopolar, Guide XT: directional	Electrode capacitance accounted for, isotropic homogenous tissue conductivity 𝜎=0.3 S/m, user adjustable: encapsulation impedance	Cathodic monophasic, user adjustable: voltage or current amplitude, pulse width, pulse frequency	Anatomical target
	Vercise Neural Navigator with STIMVIEW XT (Boston Sci.)	Non-FEM LUT: based on 5.7 μm diameter fiber (MRG model) field-axon simulations, grid geometry	Monopolar, bipolar and directional (Boston Sci. leads)	Homogeneous isotropic 𝜎=0.2 S/m, tissue encapsulation layer 𝜎=0.1 S/m	Cathodic monophasic, incorporates electrode capacitance, user adjustable: current amplitude, pulse width	Anatomical target

Regarding open-source simulation packages, Lead-DBS, provides visualization of structural atlases as well as structural and functional network connectivity maps, gives the option to choose between four methods for VTA prediction, including three non-FEM models (Kuncel et al., 2008; Mädler and Coenen, 2012; Dembek et al., 2017) and a FEM-based field-heuristic LUT model representing data from Åström et al. (2015) and Horn et al. (2019). A more recent platform release by Lead-DBS includes field-axon grid-geometry and pathway-activation capability via the OSS-DBS simulation tool (Butenko et al., 2020; Neudorfer et al., 2023). FastField (Baniasadi et al., 2020) is another open-source toolbox with only co-visualization of structural atlases and VTAs, not network connectivity maps; but it provides extremely fast computation of electric fields via precomputed FEM models for a multitude of commercial monopolar or bipolar electrode configurations and fast computation of a VTA using an empirical model fit to data from Åström et al. (2015). Linköping University has an open-source modelling tool that combines an FEM solver, ELMA (Johansson et al., 2019), with a VTA and neuroimaging platform, DBviS (Wårdell et al., 2022). This tool allows for co-visualization of VTA surfaces with structural atlases and uses electric-field-heuristic VTA methods or VTAs defined by data from Åström et al. (2015) that observes the variability due to fiber diameter and stimulus pulse width. SciRun (SCI Institute, 2016) is an open-source platform from the University of Utah that couples an FEM solver with the biophysical solver Neuron (Hines and Carnevale, 2001) for modeling field-axon simulations. This tool may be combined with structural images or network connectivity atlases and is extensively used by the Butson research group. They also have developed simplified visualization programs that can be run on a tablet for fast computation and make use of a server–client setup and computation via SciRun (Butson et al., 2013; Vorwerk et al., 2020).

As mentioned previously, the activating-function look-up-table method stemming from Butson and McIntyre (2005b) constituted the academic software Cicerone (Miocinovic et al., 2007), which was developed into the commercial tool, GUIDE, by Boston Scientific (Horn, 2017). StimVision is another academic software from the McIntyre lab that has versions stemming from Chaturvedi et al. (2013), the neural-network-based VTA predictor (Noecker et al., 2018), and most recently including the fast, pathway-activation model of Howell et al. (2019) and Noecker et al. (2021). The second version of StimVision uses the CIT168 human MRI brain atlas (Pauli et al., 2018) and the Petersen et al. axonal pathway models (Petersen et al., 2019) with patient-specific MRI information. It offers VTA estimations from a large variety of precomputed electrode configurations plus fiber activation using the Howell et al. (2019) driving-force predictor algorithm on modelled fibers in 9 general axonal pathways. It is a computationally efficient platform suited for detailed patient-specific modeling for retrospective or prospective clinical studies. SureTune is a commercial software package from Medtronic that provides VTAs in comparison to structural atlases and the ability to show fiber tracts for structural network connectivity with SureTune 4; it uses precomputed FEM data and visualizes estimated VTAs for 2, 2.5 and 3 *μ*m fiber diameters for various stimulation amplitudes and pulse widths based upon data from Åström et al. (2015) and Johansson and Zsigmond (2021). Boston Scientific continues to incorporate more capability in their commercial software packages. The latest, Vercise Neural Navigator with STIMVIEW XT, is FDA approved and provides VTA estimation with co-visualization of anatomical targets for any monopolar, bipolar or directional electrode configuration for their DBS leads. Their platform allows for user input of stimulus current amplitude and pulse width and is based on precomputed data using field-axon (grid geometry) simulations following the methods of Butson and McIntyre (2005b) with FE model parameters of 0.2 S/m isotropic heterogenous brain tissue conductivity and 0.1 S/m for the tissue encapsulation layer (Malekmohammadi et al., 2022).

## 3 State-of-the-art in clinical studies

Over the last 10 years, many clinical DBS studies have been performed that use VTA methods with neuroimaging techniques to help define target sweet spots, understand the effect of current spread as it relates to unwanted side effects, and/or determine network activity to better define the mechanism of action, for example. This section details a sample of the insights gained by the community as a function of neurological disorders by such studies.

### 3.1 Parkinson’s disease

Since Parkinson’s disease (PD) was the first to be FDA approved for DBS therapy, this disease has ostensibly the most studies for it regarding DBS effectiveness, and thus an interesting progression of model-informed insight can be seen. An early study by Maks et al. showed that through patient-specific VTA simulation with methods stemming from Butson and McIntyre (2005b) and Butson et al. (2007) a region of the dorsal STN including white matter tracts dorsal to that region was correlated with improvement in overall motor symptoms based upon the Unified Parkinson’s Disease Rating Scale (Maks et al., 2009). Alberts et al. and Frakenmolle et al. then showed decent propensity for using VTA-modeling for prospective DBS programming (i.e., stimulus amplitude and pulse width) via small-cohort studies that assessed patient therapeutic scores with and without VTA-based programing settings maximized for overlap with the regions found in Maks et al. (Alberts et al., 2010; Frankemolle et al., 2010). Both of those studies also addressed reduced cognitive function as a side-effect of non-optimal stimulation. Other studies used VTA overlap with anatomical structures to assess verbal fluency in STN DBS (Åström et al., 2010; Mikos et al., 2011) and with DBS in the globus pallidus (GP) (Dietz et al., 2013).

With the addition of more sophisticated combinations of VTA and neuroimaging, targets were better defined, and networks associated with optimal therapy and side effects were identified. The first study that defined the method of probabilistic stimulation mapping of a cohort of patient-specific VTAs, albeit made from normative structural atlases (Butson et al., 2011) reinforced the results of Maks et al.; and they defined further specificity between improvements in rigidity and bradykinesia as a function of spatial regions in the STN. Vanegas-Arroyave et al. performed a seminal study that used DTI tractography and VTA to give network-based information on the mechanism of action of therapeutic STN DBS (Vanegas-Arroyave et al., 2016). They used patient-specific tractography from 3 T MRI of all 22 patients and the simple, non-FEM VTA method of Mädler and Coenen (2012) and surmised that the dentato-rubro-thalamic tract, zona incerta and/or pallidothalamic tract directed towards the thalamus contributed to clinically effective DBS based upon the therapeutic window established during monopolar review. Another study assessing structural connectivity via tractography and VTA looked for the network effect of speech disturbances in STN DBS (Mahlknecht et al., 2017). Their VTA and neuroimaging modeling done via SureTune (Medtronic, MN) was used to depict overlap of corticospinal and corticobulbar tracts. The corticospinal and corticobulbar tracts in this study were derived from patient-specific tractography and then averaged across the group. This information was leveraged to determine that the activation of the internal capsule was inversely correlated with the resting motor thresholds of the contralateral orbicularis oris muscle and first dorsal interosseus muscle, in the face and the hand, respectively (Mahlknecht et al., 2017). Horn et al. then perfromed a study of both structural and functional network-connectivity seeded by VTA for STN DBS (Horn et al., 2017). They used the FEM-based VTA model available in Lead DBS at a threshold value of 0.2 V/mm with tractography either from a normative connectome generated from a large database of healthy subjects or a normative connectome generated from a database of 90 PD patients matched for sex and age. They showed that connectivity results were similar with both the healthy and PD connectomes and that effective STN DBS largely echoed the results found in Vanegas-Arroyave et al. More specifically, the supplementary motor area (SMA), anterior cingulate, and medial prefrontal cortex were correlated with effective DBS for overall motor improvement, while M1 was negatively correlated. Their study also paved the way for probabalistic network-based atlases made from large populations in retrospective studies. Importantly, their study also showed that the connectivity profiles (or network-based atlases) derived from one cohort could be used to predict clinical efficacy in independent cohorts. Around the same time, Akram et al. perfromed a study that used SureTune-derived VTAs and a voxel-wise statistical approach for a target-based probabilistic stimulation map and structural connectivity analysis for STN DBS. The tractography was patient-specific and they surmised the following details: the central portion of the superior STN is most effective for tremor, while stimulation in medial and posterior areas, within the superior portion, gives highest improvements in bradykinesia and rigidity; also connectivity to M1 appears to predict improvement in tremor, SMA predicts improvement in bradykinesia, and both SMA and the prefrontal cortex (PFC) predicts improvement in rigidity (Akram et al., 2017). Of note, Dembek et al. later proposed a “sweet spot” slightly more dorsal and lateral than that of Akram et al. using a similar voxel-wise statistical approach for probabilistic mapping with VTA estimations via the Lead DBS framework using a FEM-heuristic approach with heterogenous tissue conductivity for white and grey matter and an electric field threshold value of 0.2 V/mm and was also able to cross-validate this model in a completely independent second cohort (Dembek et al., 2019). During cross-validation, the model was found to explain 20% of the variance in motor score improvement in the independent cohort (*p* < 0.001). Another study used non-atlas-based patient-specific VTA estimation for voxel-wise probabilistic stimulation maps of DBS efficacy for PD (Malaga et al., 2021). Using true patient-specific MRIs for structure segmentation and anisotropic conductivity they calculated VTAs via electric-field threshold values as a function of stimulation parameter given by Åström et al. (2015) and mapped optimal stimulation locations to be regions dorsomedial to the STN, near the posterior half of the nucleus.

Lin et al. performed a study with patient-specific tractography and non-FEM VTA via Lead-DBS and used machine learning (random forest classifiers) to characterize specific connectivity features with DBS outcome (Lin et al., 2020). They found the thalamus, hippocampus, pallidum, M1, SMA, and the superior frontal gyrus (SFG) all corresponded with effective DBS contacts. Additionally, the concept of using machine learning to classify and/or provide a predictive model for DBS effectiveness based on VTA-based neuroimaging data is a topic of interest and has been implemented for STN DBS in another recent study (Chen et al., 2022). Other recent work use VTA-seeded connectivity maps to take closer look at side effects including depression in STN DBS (Irmen et al., 2020), stimulation-induced dysarthria (SID) during STN DBS (Dayal et al., 2020), and SID in GPi/GPe DBS (Tsuboi et al., 2021).

There are fewer studies using pathway activation models in the literature, but they pose to give more nuanced information on the mechanism of action. Butenko et al. suggest using PAM to define a profile of pathways whose balanced activation alleviates the profile of symptoms (Butenko et al., 2022). Their study results show that is not key to activate/modulate a single specific tract (such as the hyperdirect pathway alone) but instead a specific array of tracts connecting or passing the STN including the pallidothalamic projections: the ansa lenticularis and lenticular fasciculus (Butenko et al., 2022).

### 3.2 Essential tremor

DBS programming for tremor suppression in essential tremor (ET) is one of the most straightforward procedures in neuromodulation. There is direct visual feedback for tremor suppression that responds in real time (on the order of seconds) to guide DBS programmers. However, the challenge in DBS for ET lies in maximizing tremor suppression while minimizing stimulation induced side effects. Here, several groups have tried using VTA models to solve this task. For example, similar to Tsuboi’s characterization of stimulation induced dyskinesia in PD, Petry-Schmelzer et al. sought to determine the brain network fingerprint of stimulation induced dysarthria in ET patients as well as build a predictive model for stimulation related speech intelligibility after thalamic DBS (Petry-Schmelzer et al., 2021). FEM-based VTAs were used to evaluate structural connectivity using a discriminative fiber tract analysis within a normative connectome. The model was able to demonstrate that 64% (*p* < 0.001) of the variance resulting from stimulation induced speech unintelligibility could be explained by the identified fibers (Petry-Schmelzer et al., 2021). The authors also found that the majority of stimulation induced dysarthria was associated with motor cortex or cerebellar modulation. This study highlights the capability of using VTA to create fiber filtering algorithms that can identify brain networks implicated through stimulation.

Target-based probabilistic stimulation maps derived by VTA modeling have shown potential in reducing the clinical programming and optimization for DBS in ET and in assessing optimal targets for side-effect suppression. Åström et al. explored how VTA models could be leveraged to predict effective electrode contacts when specifically targeting the caudal zona incerta (Åström et al., 2018). Åström et al. created atlas-based FEM-based VTA models coupled with an axon cable model in an isotropic, homogenous tissue medium (Åström et al., 2018). The VTA-based model was able to predict the exact clinical contact 60% of the time and match within the top 2 options 83% of the time. In a recent study, Malaga et al. show that added patient specificity by using patient MRI DTI data for defining the microstructure of the brain regions instead of atlases and unique DTI-informed tissue conductivity to generate the VTAs for probabilistic stimulation mapping helped explain undesirable side effects (Malaga et al., 2023). They used an FEM-heuristic VTA model with threshold of 0.2 V/mm and found that the patient-specific structure-based VTA performed better than atlas-based VTA prediction in explaining sustained paresthesia. 94% of the patient-specific VTAs overlapped with sensory thalamus estimates compared to only 74% of the atlas-based VTAs.

VTA-based predictive models have also been utilized to refine surgical precision and predict therapeutic outcomes. Middlebrooks et al. employed FEM-derived VTA models within a normative connectome framework to derive structural connectivity indices for 97 ET patients undergoing unilateral thalamic DBS (Middlebrooks et al., 2021). These indices, derived from VTA modeling, facilitated the creation of a unique spatial connectivity “fingerprint” for each subject, which was then applied in a leave-one-out cross-validation scheme to prognosticate the percentage of tremor reduction post-DBS. The connectivity “fingerprint” demonstrated a significant correlation with tremor suppression (*R* = 0.41, *p* < 0.0001) and robustly predicted outcomes in a completely independent cohort of 14 ET patients (*R* = 0.59, *p* = 0.028). Subsequent analysis of the model indicated that the neural regions most indicative of tremor suppression coincided with the dentato-rubro-thalamic tract (DRTT). The authors concluded that the DRTT can be leveraged as an anatomic region of interest for future tremor intervention.

### 3.3 Epilepsy

Medication refractory generalized epilepsy is another challenging field that has benefited from VTA analyses to guide neuromodulation therapy. Although many brain networks such as the default mode network (DMN) have been implicated, identifying a common epileptic origin remains elusive and difficult (Cataldi et al., 2013). Previous electrophysiology studies have suggested that epileptic pathogenesis may originate from the centromedian nucleus of the thalamus (CM) and be modulated by the anterior nucleus of the thalamus (ANT). DBS of these targets have shown promise in clinical trials but there remains significant variability in seizure outcomes (Salanova et al., 2015). Several studies have employed VTA based models to test these hypotheses and explain the variance seen in clinical studies. Middlebrooks used FEM-based VTAs (using 0.2 V/mm threshold for activation following Horn et al., 2017) from 6 patients with refractory epilepsy as seeds for a functional connectivity analysis within a normative connectome to try to explain the variability in seizure response after ANT DBS (Middlebrooks et al., 2018). This method revealed that 3 patients who were strong responders, defined as seizure frequency reduction by at least 50%, had much stronger connectivity to the DMN compared to the other 3 patients who did not have a strong response after ANT DBS. Similarly, Torres Diaz et al. conducted an FEM-based VTA study (using 0.2 V/mm threshold for activation following Horn et al., 2017). They incorporated both structural and functional connectivity mapping in 10 patients with generalized epilepsy who received CM DBS (Diaz et al., 2021). By using the VTA as a seed for both patient-specific diffusion MRI and normative resting state fMRI, Torres Diaz et al. identified a well-delineated network comprised of sensorimotor and supplemental motor cortices, the brainstem, and cerebellum that correlated with the greatest seizure reduction. Interestingly, both structural and functional connectivity analyses converged to a similar network, illustrating how VTA-based network analyses can refine the region of interest for targeted neuromodulation. However, the use of non patient-specifc VTA modeling in these studies may not accurately capture the axonal modulation in these dense thalamic nuclei and this effect may be further amplified using a normative connectome. Charlebois addressed this issue by employing an enhanced VTA model using FEM based upon electrical conductivity derived from patient specific DTI sequences and incorporating an encapsulation area around the lead. Additionally, they utilized activation function thresholds that were obtained from biophysical field axon models which are derived from stimulation parameters (following methods in Butson and McIntyre, 2007 and Duffley et al., 2019). These VTAs were used as seeds for structural connectivity analyses across both a normative connectome and patient-specific connectome in 22 patients implanted with an RNS neurostimulator (NeuroPace, Mountain View, CA) (Charlebois et al., 2022). Through this technique, Charlebois found that there was no significant correlation between the normative connectivity map and seizure reduction (*r* = 0.28, *p* = 0.09), but there was a significant correlation between the patient-specific connectivity map and seizure reduction (*r* = 0.74, *p* < 0.0001). These studies provide an excellent showcase of the impact VTA and tractography model selection has on connectivity results and how factors such as disease pathology and network complexity may play a role in how connectivity studies should be designed in the future.

### 3.4 Dystonia

While DBS for dystonia can be incredibly effective, the time course for improvement can be highly variable and up to 25% of patients may be non-responders (Isaias et al., 2009; Volkmann et al., 2012). Although there is a consensus to target the motor region of the GPi, the degree of variability in patient outcomes necessitates an urgent need to better understand neuromodulation in dystonia. Cheung combined FEM-based field-axon VTA models (following Butson and McIntyre, 2007) into a target-based probabilistic stimulation atlas to map the optimal regions of stimulation in GPi DBS for dystonia (Cheung et al., 2014). Patient-specific VTAs were transformed into normalized template space and thresholded to include only voxels that provided at least 75% improvement and were shared by at least 75% of the cohort. This technique identified a region in the center of the posterior portion of the GPi that was associated with the greatest dystonia improvement. This study demonstrates how VTAs can be used to guide DBS targeting from a broad spatial perspective and characterize general anatomic trends/relationships. As the authors cautioned, however, this technique was designed to simulate the region of influence from DBS therapy and makes no assumptions regarding disease specific pathology or patient specific connectivity. It is simply aimed to define the anatomic regions most frequently modulated by DBS. Reich et al. expanded on this approach and used FEM-based VTAs (via SureTune), methods from Åström et al. (2015), which used homogenous isotropic conductivity as well as a single cable axon model to create a target-based probabilistic stimulation atlas from a much larger cohort of 105 patients (Reich et al., 2019). This atlas was also transformed into normalized template space but identified a different region compared to Cheung et al.—the ventroposterior GPi as well as the surrounding white matter tissue. Although the authors offer several explanations for these differences, including limitations of image processing and the use of voxel-wise statistics rather than a threshold, this also highlights the limitations of FEM-based VTAs being used as a static approximation of the DBS electric field and converting this information into a 3D volume. The authors rightly point out that using VTAs in this manner would highlight a “volume” of neural tissue rather than a “target.” However, later work by the same group (Soares et al., 2021) using predictions based on the same computational methods corroborated the ventroposterior GPi as the target sweet spot for isolated dystonia and combined dystonia patients. Their study also stated that only 32% of the variance in patient outcomes could be predicted by their model and cautioned that this type of model alone would not be sufficient for clinical prediction.

### 3.5 Obsessive compulsive disorder

VTA-seed-based connectivity maps have also been used in OCD DBS to guide the target selection and DBS programming optimization processes. As OCD is a heterogenous condition and thought to be a neurologic disorder that results from multiple overlapping dysfunctional neural networks, connectivity mapping has emerged as a useful to tool to highlight regions of interest that are most associated with clinical improvement. As most clinical trials in OCD DBS typically have less than 20 patients, many connectivity studies employ the use of normative connectomes as a foundation for their analysis. In scenarios where the study population is limited, the strength of VTA seeding within a normative connectome is highlighted as patient-specific tractography of a small cohort would otherwise be too noisy to interpret. Baldermann and Li both used FEM-based VTA (Lead-DBS and/or SureTune) structural connectivity analyses within a normative connectome to illustrate that specific connectivity profiles could be generated that predict clinical outcomes (Baldermann et al., 2019; Li et al., 2020). Both studies detected relevant fiber tracts within the anterior limb of the internal capsule that led to the identification of a unified hyper direct pathway connecting the dorsal anterior cingulate cortex to the anteromedial subthalamic nucleus. Li further challenged the generalizability of this model by cross-predicting clinical outcomes from completely independent cohorts from other institutions. Gadot combined the results of the FEM-based VTA (via Lead-DBS) connectivity with discriminative fiber tract analysis to predict the clinical outcomes of another independent cohort of 10 patients in a rank-based fashion (Gadot et al., 2023). Gadot’s model was able to accurately predict the ranked order of improvement in the 10 patients (Spearman correlation *r* = 0.75, *p* = 0.013), demonstrating the utility of VTA based models even within normative connectomes.

## 4 Discussion

### 4.1 General themes and limitations

Within specific neurological disorders, the use of VTA-informed neuroimaging to suggest sweet spots for DBS targets and/or activation of connected fiber tracts that innervate distant cortical and subcortical regions has advanced the field of DBS neuromodulation. Generally, the field has adopted the use of retrospective clinical data to make VTA-informed models of target or network-based maps that quantify the probability of effective patient outcome (i.e., probabilistic stimulation atlases) to provide more insight on the condition and in some cases to predict patient efficacy in independent patient cohorts based on lead placement and DBS parameters. However, despite the added benefit these data-driven models have provided, the models cannot fully explain the degree of variability in clinical outcomes.

Limitations to the modeling techniques that contribute to error come from uncertainties in both the neuroimaging side of modeling as well as the VTA/pathway activation side. On the imaging side there is inherent error in the following methods: co-registration of DBS leads from pre-and post-operative brain MRI or CT scans, warping of established atlases of target brain structures to individual patients, using a normative structural atlas or connectome versus patient-specific microstructure and fiber tract information, and DTI-based tractography estimation (e.g., probabilistic or deterministic). On the VTA side, one obvious source of error is that the volume defined by the VTA is unphysical as the true spatial nature of the axon pathways are not included. Gunalan et al. showed that when comparing pathway-activation models to VTA models that calculated fiber-tract overlap to define structural network connectivity, there was a large discrepancy between the results (Gunalan et al., 2018). Further, if the VTA is assumed to provide a good estimate of spatial activation, then differences within the parameters chosen for FEM-based VTAs can also contribute to variation. The parameter choices include using isotropic, anisotropic, homogeneous, and/or heterogenous tissue conductivities (whose values for specific materials may vary between studies), modeling a high resistive encapsulation region around the lead or not, modeling the capacitive effects of the electrode interface and time nature of the stimulus pulse trains including frequency or not, and the choice to use heuristic threshold levels or one derived or defined by axon models add variation and error in the final model.

Regarding FEM-based models, the choice of how to model the brain tissue with respect to electrical conductivity has seen much variation in the literature. Early on, it was shown that inclusion of a high resistivity tissue encapsulation area adjacent to the DBS lead in isotropic tissue models made significant changes to the resulting VTA (Butson et al., 2006). In later studies, tissue anisotropy was evaluated via pathway activation models of neural axons. Chaturvedi et al. showed that anisotropy of the tissue outside the encapsulation region, as defined by local DTI-informed conductivity tensors will further influence (i.e., a reduction of the spatial extent of axonal activation), which more closely matched clinical estimates of stimulation (Chaturvedi et al., 2010). Additionally, Howell and McIntyre compared models of heterogeneity (i.e., white and grey matter), anisotropy, and frequency effects to isotropic homogeneous conductivity models and found that inclusion of anisotropy had the largest effect followed by heterogeneity (Howell and McIntyre, 2016). They also showed that different methods to compute anisotropic conductivity tensors, with methods that did or did not incorporate influence of a measured load impedance (i.e., electrode interface effects), resulted in differences in neural activation prediction. Moreover, dielectric dispersion (i.e., modeling a complex conductivity, σ+jωε ) had the smallest effect (<1% mean average difference) within anisotropic models. In their study, a tissue encapsulation region was present in the isotropic cases but not in the others. In a recent study, Liu et al. show how patient-specific anisotropic tissue conductivity in VTA-based models contribute to significant patient variation across a cohort of 40 patients as well a significant deviation from isotropic conductivity-based models (Liu et al., 2024). Out of the three commercial software platforms listed in Table 1, all use homogeneous isotropic brain tissue with three different values for conductivity and only two (Guide and Vercise Neural Navigator) incorporate a tissue encapsulation layer. One more aspect to point out, the complexity of the volume conductor of the head model and its grounding scheme for monopolar stimulation can also contribute to differences in voltage field calculations and thus VTA or pathway activation results especially if the overall impedance value measured via the IPG is used to tune the model (Grant and Lowery, 2009).

Regardless of all of these model-specific variabilities, one aspect to point out is that if neuroimaging methods use average information from population groups and normative structural atlases or connectomes for probabilistic maps, we believe that the extent of VTA specificity (e.g., FEM or not, field-axon or heuristic threshold, etc.) will have less of an effect on the variation of the outcome prediction by such a model, as the variability due to averaging across a population may be larger than variability introduced in VTA methodologies. Moreover, modeling studies that use atlases for determination of DTI-based conductivity tensors in FEM VTA or PAM predictions in retrospective studies could erroneously alter the local conductivity for a specific patient and thus add error to the group-based probabilistic map. For true patient-specific applications, the choice of the mentioned model parameters will likely contribute a great deal to the accuracy of the VTA or pathway activation prediction.

Regarding the use of VTA-informed neuroimaging methods (i.e., target vs. connectivity-based), it is interesting to see that within the different disorders, some modeling paradigms are more consistently used than others. For PD and ET both target-based and network-based probabilistic stimulation mapping have been used by the community and some combine both methods (Akram et al., 2017; Tsuboi et al., 2021). On the other hand, recent work on dystonia has been focused on target-based analyses and not network/connectivity to fine tune the sweet spots for stimulation in the GPi, leading to question if differences in target results by different groups could be resolved with the addition of network-based mapping used in conjunction. And recent studies for epilepsy and OCD have only used VTA-based network/connectivity mapping to provide insight. Pathway activation models that provide information on network connectivity are seen even more seldom in the literature, potentially because of the computational complexity of the method.

### 4.2 Future of neuromodeling for DBS

Understanding the brain within the context of neurological disorders and neuromodulation therapy is clearly challenging and subject to many individual idiosyncrasies; however, it is impressive how VTA-based neuromodeling, regardless of the VTA method, has been able to provide added detail for target sweet spots, regions of fiber tracts that correlate with unwanted side effects, and more insight into the mechanism of action for effective DBS with much consensus between multiple studies. One of the key features that is apparent in the literature is the notion that predictive models can be garnered from population/group results of retrospective studies for a particular disorder. The extent to which those models (probabilistic stimulation atlases, target-and/or network-based) need to be fine-tuned for a more precise predictive model is a question yet to be answered. Recent work by Johnson et al. show that the added specificity of pathway-activation models with patient-specific structural connectivity analysis yielded robust metrics for prediction of patient outcome for GPi DBS for Tourette syndrome, which is a disorder that has had high variability of patient responses to DBS treatment (Johnson et al., 2021). Also, Hollunder et al. suggest a paradigm shift in how group-based atlases are used for the end patient (Hollunder et al., 2022). They suggest the creation of network-based atlases/templates from population studies for the disease symptom (e.g., tremor, rigidity, cognitive flexibility, anxiety) rather than just the disease, and then those templates can be added together to create guidance for personalized DBS target and stimulation. And the recent work by Malaga et al. promotes the use of patient-specific microstructure (vs. atlas-based) in addition to patient-specific tissue conductivity to create better informed target-based probabilistic stimulation atlases (Malaga et al., 2021, 2023).

Modeling of tissue activation coupled with neuroimaging techniques form the computational basis for predictive models generated from group retrospective studies as well as for establishing the patient baseline data for personalized medicine that makes use of predictive models. The degree to which the predictive model is accurate for an individual should be highly influenced by the level of patient accuracy of the data from the tissue activation/modulation model and the neuroimaging methods (e.g., for generation of brain structures and fiber pathways). New retrospective studies that will generate target-based or network-based probabilistic stimulation maps might indeed benefit from more accurate patient-specificity for brain structures and fiber tracts. Innovation in DTI-based methods would serve to increase model accuracy. If patient-specific parameters are used for target-based studies, then the VTA method should also be most accurate/specific and employ field-axon simulations, which are dependent on stimulation parameters, rather than field-heuristic or non-FEM VTA models. Moreover, there is a lack of clinical comparative studies that evaluate the influence of PAM vs. VTA-based network connectivity mapping, for example. The field would be greatly served if more comparative studies using different neural activation/modulation approaches were performed.

Furthermore, it is essential to validate the strength and general applicability of these models through carefully planned and extensive prospective clinical trials. Conducting such trials is vital for establishing credibility with clinicians, which in turn, will promote the incorporation of these models into routine clinical practice.

## Author contributions

EP: Conceptualization, Visualization, Writing – original draft, Writing – review & editing. CF: Conceptualization, Writing – original draft, Writing – review & editing. DP: Writing – original draft. JC: Writing – original draft. AP: Writing – original draft. HS: Writing – original draft. JW: Conceptualization, Writing – review & editing.
