# Corrigendum: Modeling the volume of tissue activated in deep brain stimulation and its clinical influence: a review

**DOI:** 10.3389/fnhum.2024.1434402

**Published:** 2024-05-31

**Authors:** Erin E. Patrick, Chance R. Fleeting, Drashti R. Patel, Jed T. Casauay, Aashay Patel, Hunter Shepherd, Joshua K. Wong

**Affiliations:** ^1^Department of Electrical and Computer Engineering, University of Florida, Gainesville, FL, United States; ^2^College of Medicine, University of Florida, Gainesville, FL, United States; ^3^Department of Neurology, Fixel Institute for Neurological Diseases, University of Florida, Gainesville, FL, United States

**Keywords:** volume of tissue activated, VTA, deep brain stimulation, DBS, neuroimaging, probabilistic stimulation atlas, connectivity maps

In the published article, there was an error in Section **2 Calculating the VTA**, subsection 2.2.3 Field-axon- pathway activation models, 2^nd^ paragraph. The sentence erroneously stated:

“Since multi-compartment axon models are computationally intensive, subsequent work by Howell and McIntyre developed a linear approximation to the multi-compartment axon model that allowed for much faster prediction of FEM-informed pathway-activation models (Howell and McIntyre, 2016).”

The corrected sentence appears below:

“Since multi-compartment axon models are computationally intensive, subsequent work by Howell et al. (2019) developed a linear approximation to the multi-compartment axon model that allowed for much faster prediction of FEM-informed pathway-activation models.”

The authors apologize for this error and state that this does not change the scientific conclusions of the article in any way. The original article has been updated.

